# Design and operation of high-power permanent magnet speed regulator used in industry

**DOI:** 10.1038/s41598-023-29187-7

**Published:** 2023-02-14

**Authors:** Yimin Lu, Xiangdong Wang, Chunlai Yang, Long Shao, Hui Zhu, Aike Wang, ManMan Xu

**Affiliations:** 1 School of Mechanical Engineering, Anhui Polytechnic University, Wuhu, China; 2 Wuhu Magnetic Wheel Transmission Technology Ltd., Wuhu, China

**Keywords:** Electrical and electronic engineering, Mechanical engineering

## Abstract

A high-power permanent magnet speed regulator is applied to a cooling water pump for conserving energy during the steel production in Magang (Group) Holding Co., Ltd. The designed setup of high-power permanent magnet speed regulator with a mobile base is shown in this manuscript, and the magnetic eddy under the different meshing area between driving and driven shafts has been simulated. And estimation indicates that the magnet speed regulator-controlled cooling water pump can save electric energy by 22%, about 1,756,400 kW·h per year, compared to the traditional valve-controlled pump, and the waste heat generated by this setup is below 5 ten-thousandths of the shaft power. Meanwhile, the permanent magnet speed regulator has a much lower vibration because of this non-contact way between the driving and driven shafts.

## Introduction

Compared with the traditional gear box, the permanent magnet speed regulator, which is based on the magnetic eddy due to the relative motion between the permanent magnet and the conductor, has several advantages such as higher energy efficient, stronger reliability, easier installation, lower cost, and soft start of motor^1,2^. The new technology for permanent magnet adjustable speed is used for the motor to control its speed and save its energy, which is benefit to the emission reduction. Therefore, more and more attention has been paid by the researchers in the industrial field.

Development of the disc-type permanent magnet speed regulator never stops, since it was proposed in 1990s^3,4^. In recent years, researches on the permanent magnet speed regulator were carried out not only in the items of the model and simulation for the basic analysis^5,6^, but also in the application with structure improvement in industry^7,8^. The virtual equivalent line method was developed to solve the end-effect on the magnetic field distribution in the air gap region of permanent magnet speed regulator. Through calculation of the static air gap flux density, it was found that the end-effect compensation function calculated based on the model was highly consistent with the result calculated by finite element method^9^. According to model for 3D transient eddy field in some research, the substitution of the copper disc by an aluminum disc could improve the speed governing stability of the permanent magnetic coupler^10^. A fast and accurate 3D modeling method was proposed to evaluate the electromagnetic performance of machines with axial flux permanent magnet under no-load conditions. The computed results of local field density, electromotive force, and cogging torque for the disk speed regulator were in very good agreement with the experimental measurements^11^. A novel flux-weakening control strategy with fast transient current response is designed to facilitate the flux-weakening control application on electric vehicle, and the simulation and experimental results exhibited that the proposed strategy could achieve the fast torque response and also have the capacity to reduce the torque fluctuation of the stable state^12^. Structure of 250 kW permanent magnet governor was advanced to improve the heat conduction, guaranteeing the steady as well as reliable operation of this equipment^13^. Halbach array, a kind of special permanent magnet array, was tried in the permanent magnet coupler, and both the simulation and test showed higher efficiency in the axial speed regulator^14,15^. In addition, Halbach array was also used in the separators to improve the separation efficiency^16^. Relatively systematic researches on permanent magnet speed regulator were also shown in the references^17–19^.

In this manuscript, 450 kW permanent magnet speed regulator used for cooling water pump in steel production is shown. N–S pole array structure is used in this speed regulator, and the magnetic field and current density in the conductor driving draft induced by the magnet driven draft are simulated based on finite element modeling (FEM) analysis. Under the motor, an automatic mobile base controlled by programmable logic controller (PLC) is used novelty to adjust the meshing area and consequentially magnetic eddy, changing the output power of the motor to keep the water flow in pipe. Measurement and calculation shows that the magnet speed regulator-controlled cooling water pump in this research can save electric energy by 22%, about 1,756,400 kW·h per year, compared to the traditional valve-controlled pump. Therefore, this kind of permanent magnet speed regulator is in a compact structure (axial pitch is only 25 cm). Furthermore, an obviously reduction of vibration is observed when the permanent magnet speed regulator is used. Meanwhile, waste heat in the setup is very low based on the theory of thermal radiation.

## Structure and simulation

**Figure 1:**
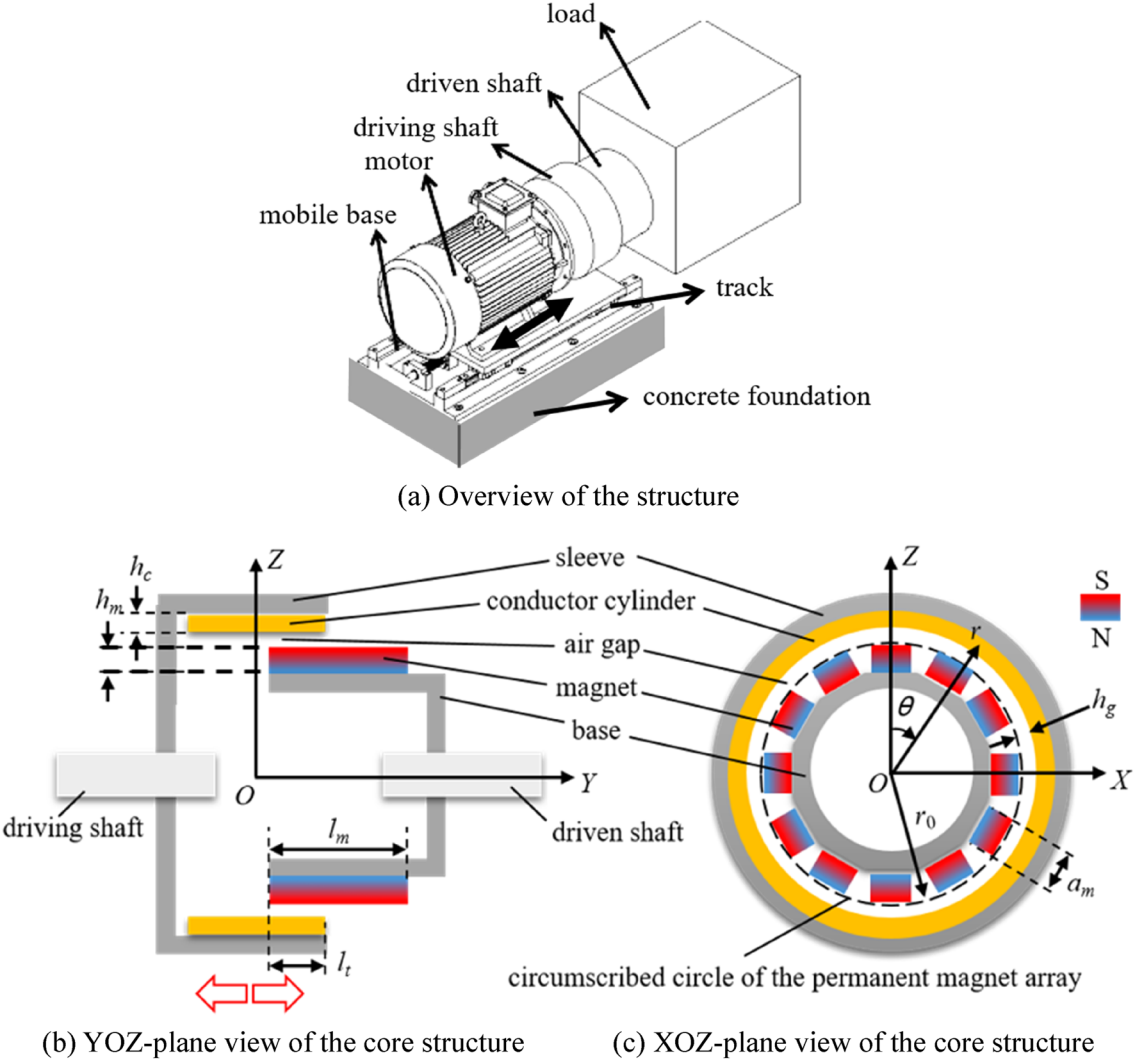
Structure of the permanent magnet speed regulator.

Based on former research and application^20,21^, the permanent magnet speed regulator, which is used to adjust the high-power cooling water pump during the steel production, is advanced to increase transmission efficiency and heat dissipation efficiency. The structure of the advanced permanent magnet speed regulator with the coordinate system is shown in Fig. 1.

As seen in Fig. 1a, this permanent magnet speed regulator includes motor, driving shaft, and driven shaft dominantly, and the motor is fixed on a mobile base with the track. The driving shaft is fixed on the motor. When the driven shaft is insert into driven shaft along with the track, meshing area between the driving and driven shafts increases as seen in Fig. 1b, enhancing the rotational resistance moment of the motor. In order to keep the rotative velocity, the motor outputs higher power. Detection, judgement, and control to the driving shaft fixed the motor are carried out by PLC.

As seen in Fig. 1b,c, 12 pieces of permanent magnet, which are used as the key component of the driving shaft, are arranged by the alternating N–S pole array on the dodecagon-type base. The copper conductor cylinder is fixed on the inner surface of the sleeve. The dodecagon-type base and the sleeve are made of steel, and also used as yoke iron to strengthen the magnetic intensity in the region between them. Thickness of the air gap *h*_*g*_ is defined as the distance from circumscribed circle of the permanent magnet array to the inner surface of the conductor cylinder. The key sizes remarked in figure are shown in Table 1. Magnet designation used in this device is N52.

**Table 1:** Size parameters of the permanent magnet speed regulator.

Size of the single magnet (mm)			Conductor thickness (mm)	Thickness of air gap (mm)	Radius of circumscribed circle (mm)
*l*_*m*_	*a*_*m*_	*h*_*m*_	*h*_*c*_	*h*_*g*_	*r*_0_
90	30	25	5	5	187.5

**Figure 2:**
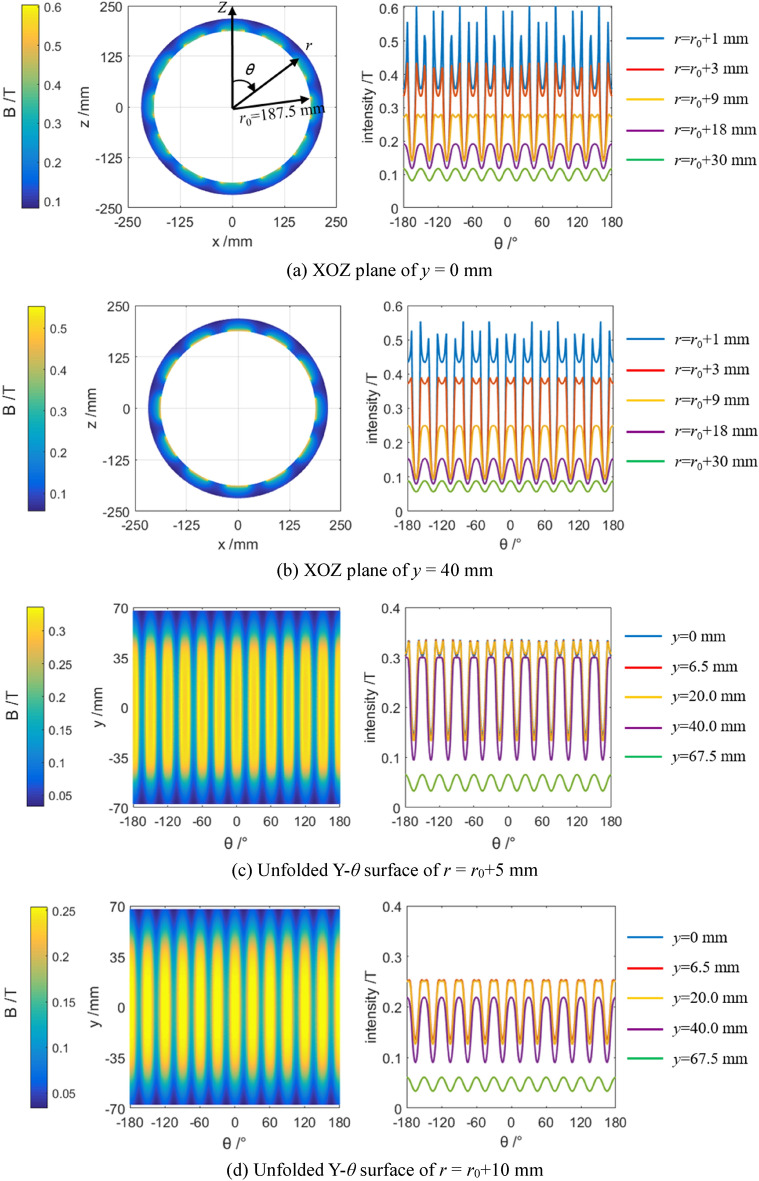
Simulated magnetic intensity *B* in the conductor.

Insert depth (*l*_*t*_) of the driving unit, which is adjusted by mobile base, can reach to the long size of the permanent magnet in *Y* axis (*l*_*m*_). The *r*–*θ* coordinate system is also introduced into XOZ plane of the Cartesian coordinate system, reducing difficulty of the calculation in simulation. According to Ampere molecular current hypothesis and Biot–Savart law^22,23^, magnetic intensity induced by the permanent magnet array has been simulated, as shown in Fig. 2.

As seen in Fig. 2, a relatively higher magnetic intensity was gain due to the yoke iron. The maximal magnetic intensity could be reached to 0.25–0.3 T in the plane away of 9 mm from the magnet array.

According to Maxwell’s equations with Faraday law^24,25^, induced current density in the copper conductor is as

1 $$\boldsymbol{J} = \, \sigma \boldsymbol{v} \times \boldsymbol{B} - \sigma \partial {\boldsymbol A}/\partial t$$

2 $$\boldsymbol{B} = \nabla \times \boldsymbol{A}$$

where *σ* is the conductivity of copper; ***B*** is the vector of induced magnetic intensity; ***A*** is an introduced vector named as magnetic potential; ***v*** is the vector of relative speed between conductor cylinder and magnet array, and it can be calculated by

3 $$\boldsymbol{v} = \, \omega { \boldsymbol{r}} = { 2}\pi sN{\boldsymbol{r}}$$

where *ω* is the relative angular frequency between conductor cylinder and magnet array; *s*, *N* and ***r*** are the slip difference, input rotation velocity, and the vector of polar axis in the *r*–*θ* coordinate system.

**Figure 3:**
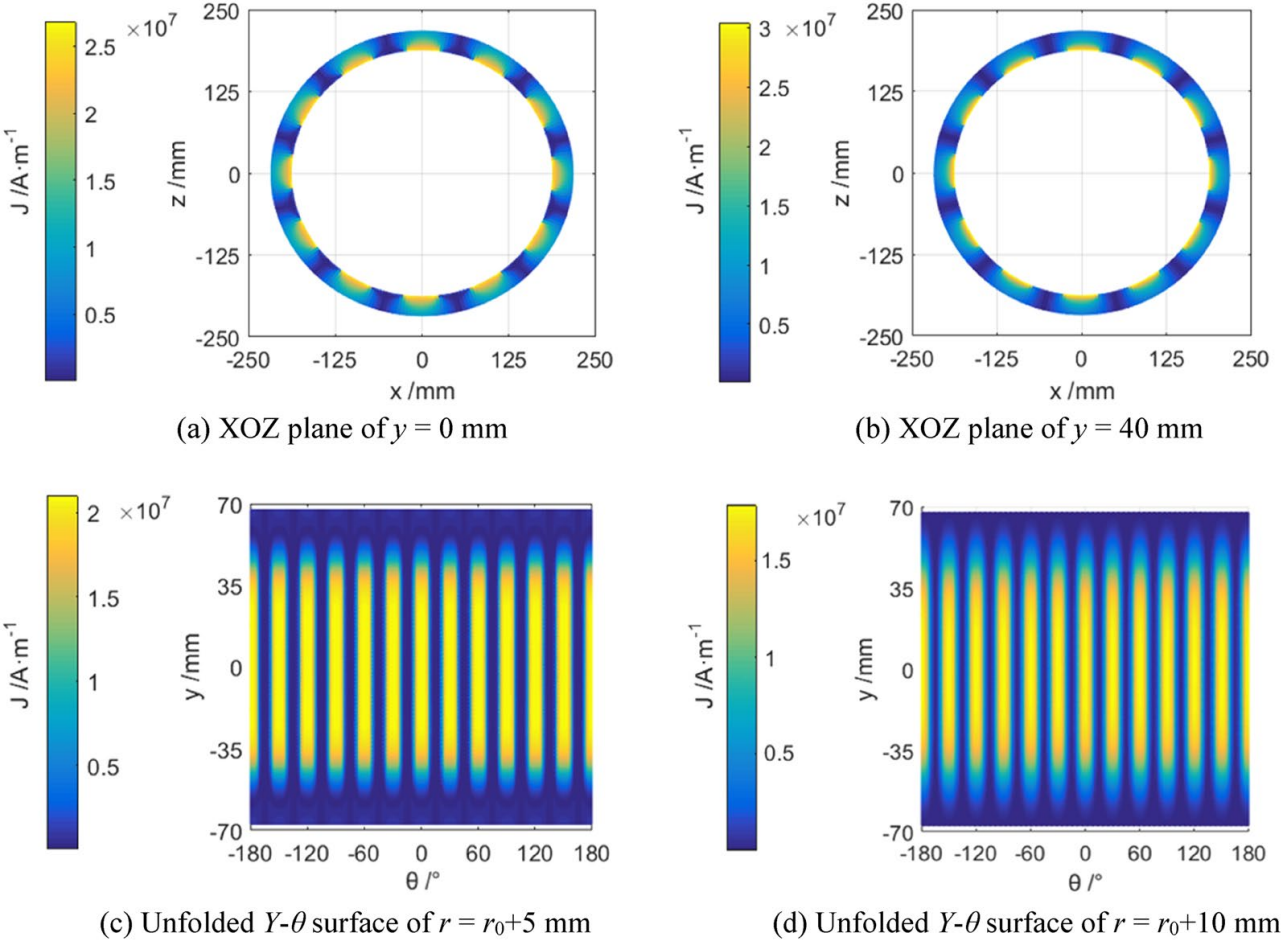
Simulated current density *J* in the conductor.

**Figure 4:**
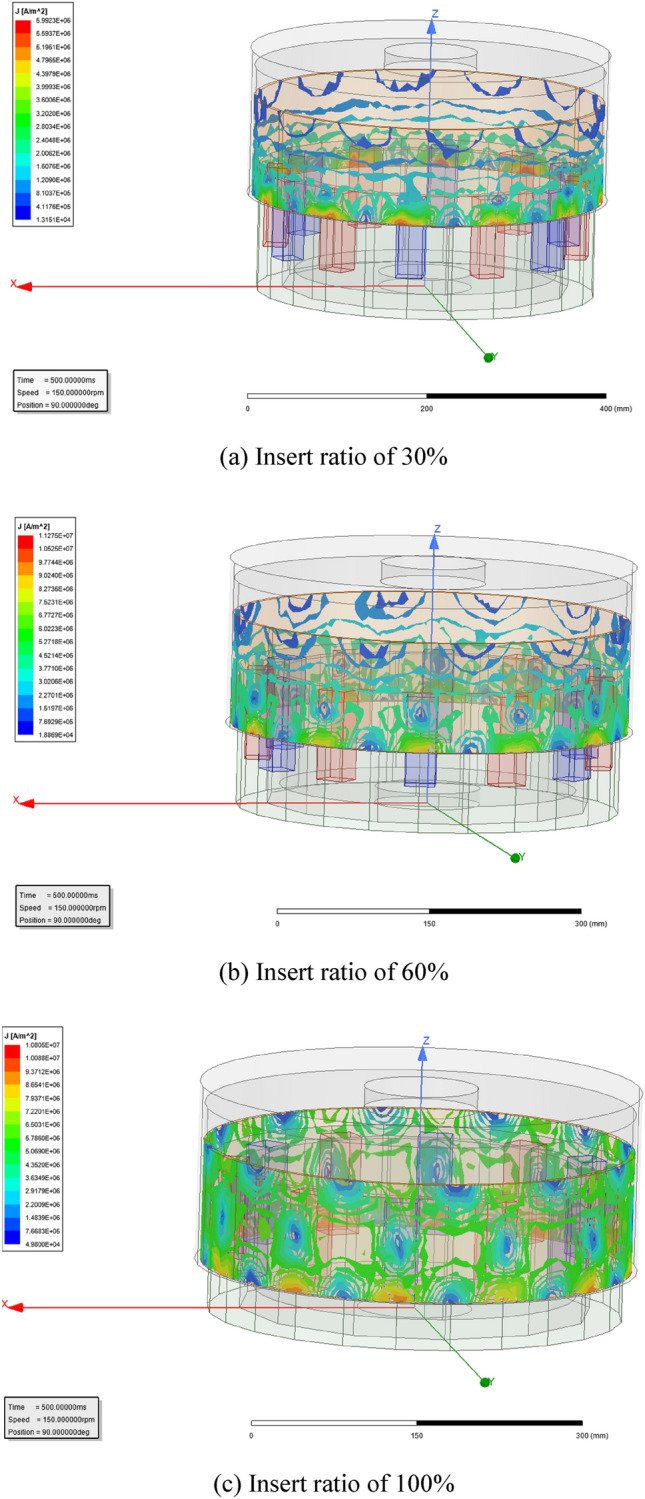
Vector-graph of magnetic eddy.

As seen in formula (3), current density ***J*** will be changed in proportional variation with the relative angular frequency *ω* or slip difference *s*. Simulated current density of the magnetic eddy induced by the relative motion between the permanent magnet array and the conductor cylinder (*ω* = 1 rad/s) is shown in Fig. 3, and their vector-graph are shown in Fig. 4c Insert ratio of 100% (Fig. 4).

As seen in Figs. 3 and 4, the 12 independent magnetic eddies can be observed clearly under different insert ratios of driving shaft. However, the surficial current intensity of magnetic eddy is influenced seriously by the meshing area due to insert ratio. Higher surficial current intensity of magnetic eddy means the larger magnetic resistance from the driven shaft against the driving shaft^16,19^. And then the latter will increase the power to keep the set rotative velocity of the motor.

## Applied equipment in the preliminary test and discussion

### Basic parameters of the high-power permanent magnet speed regulator

**Figure 5:**
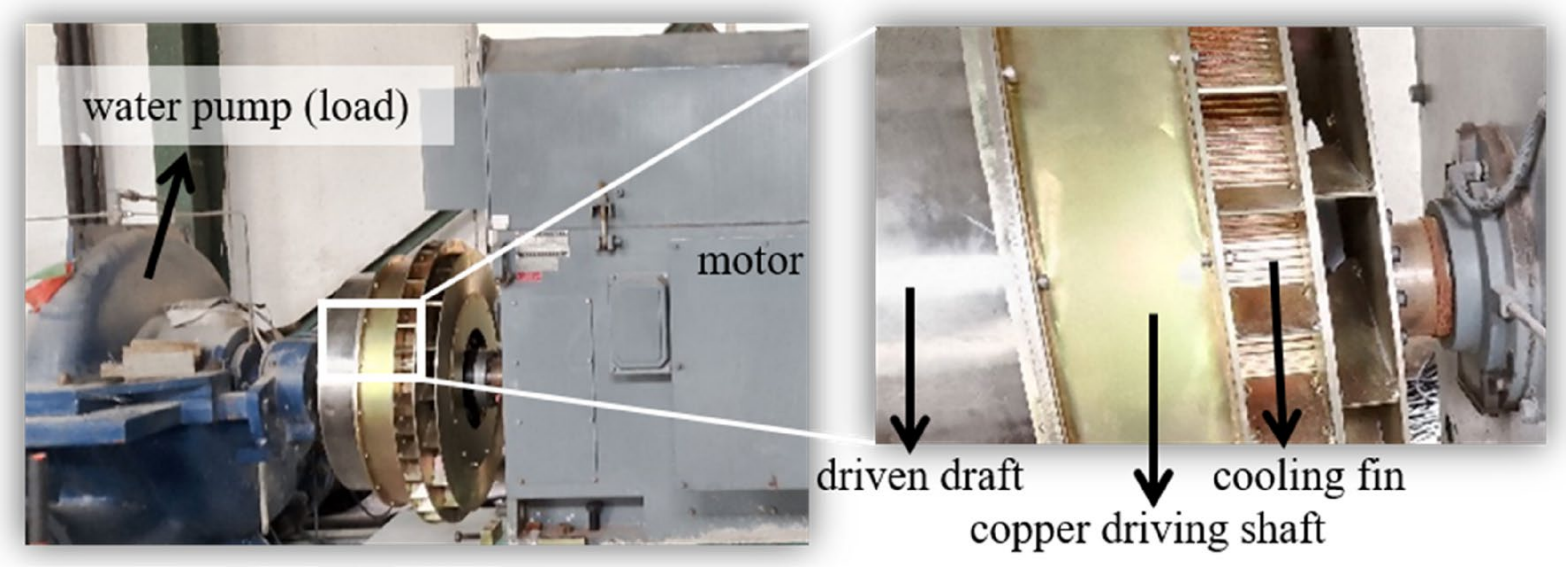
Equipment in the preliminary test.

The 450 kW permanent magnet speed regulator used for cooled water pump, as shown in Fig. 5, is tested in Magang (Group) Holding Co., Ltd. Compared with the simulated structure above, a cooling fin array is added in the applied equipment to improve the heat abstraction.

The basic parameters of motor and load (water pump) are shown in Table 2.

**Table 2:** Basic parameters of the motor and load.

Motor		Load	
Type	Y450-6	Type	500S-59
Rated power/kW	450	Rated rotative velocity/rpm	970
Rated rotative velocity/rpm	990	Rated flow/m^3^·h^−1^	2020
Rated voltage/V	6000	Rated lift/m	59
Rated current/A	52.8		
Power factor	0.86		
Running time/h	8472		

### Calculation of the saving rate

According to the fluid mechanics, the shaft power *P* is proportional to the product of flow rate *Q* and torque *H*, namely

4 $$P \propto Q \cdot H$$

And there are different relations between the parameters and the rotative velocity *n*, which are given as

5 $$\frac{{Q}_{1}}{{Q}_{2}}=\frac{{n}_{1}}{{n}_{2}}$$

6 $$\frac{{H}_{1}}{{H}_{2}}={\left(\frac{{n}_{1}}{{n}_{2}}\right)}^{2}$$

Therefore,

7 $$\frac{{P}_{1}}{{P}_{2}}\propto {\left(\frac{{n}_{1}}{{n}_{2}}\right)}^{3}$$

Table 3 show the calculated parameters, including the saving rate *R*_*s*_ that is given as

8 $${R}_{s}=1-\frac{P}{{P}_{max}}$$

where the *P*_max_ is the shaft power under the maximal rotative velocity.

**Table 3:** Performance of centrifugal load-type speed regulation.

Rotative velocity (%)	Flow rate (%)	Pressure (%)	Shaft power (%)	Saving rate (%)	Remark
100	100	100	100	0	The real saving rate will be lower than the calculated one, due to the relatively lower regulation efficiency
90	90	81	72.9	27.1	
80	80	64	51.2	48.8	
70	70	49	34.4	65.6	
60	60	36	21.6	78.4	
50	50	25	12.5	87.5	

**Figure 6:**
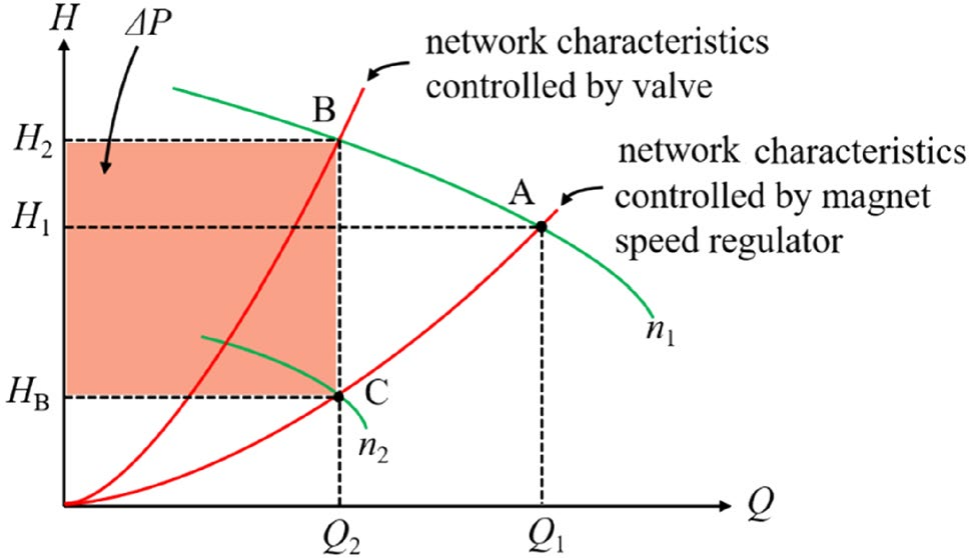
Different running properties.

Figure 6 can show the difference between the running properties of the loads controlled by the traditional valve and the magnet speed regulator in this project. When the flow *Q*_1_ decreases to *Q*_2_ due to reduced valve in the traditional valve-adjusted mode, the running condition will shift to B from A since the network resistance in pipe increases. The flow is decreased by reducing the valve, however, the rotative velocity is always kept. Therefore, the shaft power cannot be reduced.

On the other hand, the rotative velocity of the motor will be reduced by decreasing the insert depth of the driving unit in the magnet speed regulator. When the rotative velocity of the motor reduce to *n*_2_ from *n*_1_ and the flow is adjusted to *Q*_2_, the pipe also keeps a lower pressure *H*_B_. Thus, the shaft power is reduced, and the saving power *ΔP* is proportional to *Q*_2_(*H*_2_ − *H*_B_) as seen in Fig. 6. According to the statistical data, the magnet speed regulator can save power by 10%–25%. According to rated flow (2020 m^3^·h^−1^), rated lift (59 m), and minimal torque (0.45 MPa) of two motors for the cooling water in Magang (Group) Holding Co., Ltd., the rated torque is 0.58 MPa. And the saving rate is (1 − 0.45 ÷ 0.58) × 100% ≈ 22.4%. Then the two motors can save power *P*_*s*_ = 2 × 1.732 × 6000 V × 51.78A × 0.86 × 8472 h ÷ 1000 × 22.4% ≈ 1,756,400 kW·h, and the fee about 833,000 Chinese Yuan (about 120,829 dollars) per year.

### Testing of the contrast parameters

Vibration contrast before and after using the speed regulator is also shown in Table 4.

**Table 4:** Vibration contrast before and after using the speed regulator.

Vibration contrast (mm)		Day	Time	Front axle of motor			Rear axle of motor			Front axle of pump			Rear axle of pump		
				Hl	Al	Vl	Hl	Al	Vl	Hl	Al	Vl	Hl	Al	Vl
Before		1	9:00	0.6	0.9	0.9	0.9	0.9	0.9	0.8	1.1	1.2	0.8	1.3	1.3
After	Unload	51	9:00	0.8	0.2	0.4	0.6	0.4	0.3	–	–	–	–	–	–
	100% load	51	10:00	0.4	0.7	0.4	0.3	0.2	0.2	0.6	1.0	0.9	0.5	1.3	1.2
		51	12:00	0.5	0.6	0.5	0.4	0.4	0.4	0.5	1.0	0.9	0.5	0.8	0.9
		51	16:00	0.5	0.6	0.5	0.5	0.6	0.4	0.6	1.0	0.9	0.5	0.9	0.8
		51	21:00	0.5	0.4	0.6	0.7	0.7	0.6	0.5	0.9	0.9	0.5	0.9	0.9
		52	9:00	0.4	0.4	0.6	0.5	0.5	0.4	0.6	0.9	1.0	0.6	1.2	1.2
		53	9:00	0.5	0.4	0.6	0.5	0.5	0.4	0.7	0.9	1.0	0.6	0.9	1.0
Remark		Modification work was carried out in the day 1 to day 50 Hl: horizontal; Al: axial; Vl: vertical													

According to the measurement, motor vibration has a remarkable reduction after using the magnetic speed regulator, because there is no solid contact between the driving shaft (conductor) and driven shaft (magnet array), which removes the vibration amplification effect of rigid connection. Meanwhile, there is a relatively higher tolerance against the coaxiality between the driving and driven shafts also because of their non-contact state. On the other hand, a seriously accurate axis alignment whose error is below 0.05 mm is necessary in the traditional valve-controlled pump.

**Figure 7:**
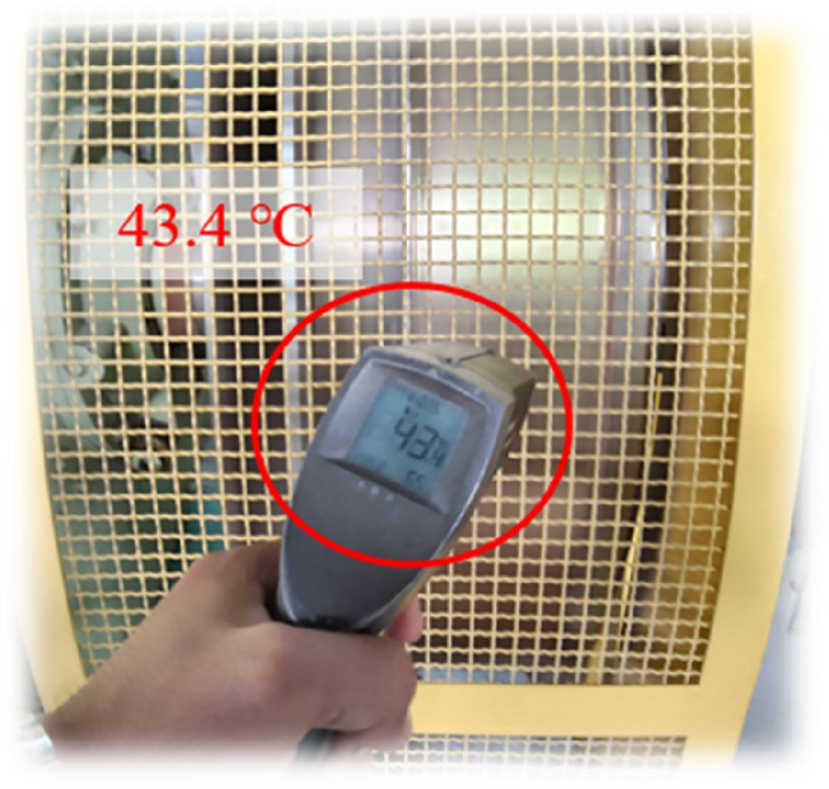
Temperature test of the cooling fin.

In addition, temperature of cooling fin was 43.4 ℃, as measured in Fig. 7, when the device runs. Waste heat emittance generated by this magnetic speed regulator can be estimated as 134 W/m^2^, if the cooling fin is supposed as a gray body with an emissivity of 0.85. Area of cooling fin is about 1.3 m^2^, and the waste heat generated by this magnetic speed regulator can be estimated as 185.9 W which is below 5 ten-thousandths of the shaft power of 450 kW.

## Conclusions

The designed and applied permanent magnet speed regulator for a cooling water pump can save large electric energy during the process of steel production by adjusting the motor power actively. An alternating N–S pole array is used as the driven shaft and a conductor used as driving shaft in the permanent magnet speed regulator. A mobile base controlled by PLC is used to adjust the meshing area and consequentially magnetic eddy, changing the output power of the motor. According to the calculation, the magnet speed regulator can save electric energy by 22% in cooling water pump, about 1,756,400 kW·h per year, compared to the traditional valve-controlled pump. Furthermore, lower vibration and much lower waste heat are generated in the permanent magnet speed regulator.

## Data Availability

The datasets analyzed during the current study available from the corresponding author on reasonable request.
